# Role of Masticatory Force in Modulating Jawbone Immunity and Bone Homeostasis: A Review

**DOI:** 10.3390/ijms26104478

**Published:** 2025-05-08

**Authors:** Yue Song, Yao Jiao, Yitong Liu, Lijia Guo

**Affiliations:** 1 Department of Orthodontics (WangFuJing Campus), School of Stomatology, Capital Medical University, Scylla alley No. 11, Beijing 100069, China; songyue@mail.ccmu.edu.cn; 2 Laboratory of Tissue Regeneration and Immunology and Department of Periodontics, Beijing Key Laboratory of Tooth Regeneration and Function Reconstruction, School of Stomatology, Capital Medical University, Tian Tan Xi Li No. 4, Beijing 100050, China; jiaoyao716@163.com

**Keywords:** masticatory force, jawbone immunity, mechanotransduction

## Abstract

Mastication exerts a significant influence on both the structural and immunological environment of the jawbone. The mechanical stress generated during chewing initiates bone remodeling through the coordinated activities of osteoclasts and osteoblasts, with these processes being modulated by immune cell responses. This review summarizes the interaction between masticatory forces and jawbone immunity, focusing on key mechanisms such as mechanotransduction in osteocytes, macrophage polarization, and the activation of T cells. The review also delves into the role of the receptor activator of nuclear factor κ-B ligand (RANKL), receptor activator of nuclear factor κ-B (RANK), and osteoprotegerin (OPG) signaling pathway, highlighting its critical function in bone resorption and immune regulation. Additionally, the review summarizes how masticatory forces modulate the immune response through changes in immune cells, particularly focusing on cytokines, and the involvement of hormonal and molecular pathways. These findings provide valuable insights into the complex interplay between mechanical forces and immune cells, with implications for bone health.

## 1. Introduction

Mastication, the process of chewing, plays a crucial role in maintaining not only the functional integrity of the oral cavity but also the structural health of the jawbone. The mechanical forces generated during chewing are essential for stimulating bone remodeling, a dynamic process that balances bone formation and resorption [1]. Beyond its biomechanical impact, mastication has significant effects on the immune environment of the jawbone. The jawbone is a unique site where immune cells are constantly exposed to mechanical stress and microbial challenges from the oral cavity, creating a complex interplay between bone cells and the immune system [2,3].

At the cellular level, osteocytes [4], osteoblasts [5], and osteoclasts [6] are key players in bone remodeling, responding to mechanical stimuli through mechanotransduction pathways [4,7,8,9,10]. These bone cells interact closely with immune cells, such as macrophages and T cells [11,12], in a bidirectional manner. Immune cells produce cytokines and signaling molecules that influence bone cell activity, while bone cells, in turn, modulate immune responses by secreting factors that affect immune cell function. Central to this dynamic interaction is the receptor activator of nuclear factor κ-B ligand (RANKL), receptor activator of nuclear factor κ-B (RANK), and osteoprotegerin (OPG) signaling axis, which not only governs osteoclast differentiation and activity but also links mechanical forces with immune modulation and bone resorption, illustrating the reciprocal influence between the skeletal and immune systems [13,14].

Understanding the relationship between mastication and the immune environment of the jawbone is critical, as this interaction affects bone health. This review aims to explore the mechanisms through which masticatory forces influence the immune response and bone remodeling, highlighting the roles of key cells and signaling pathways involved in this intricate process. By delving into these mechanisms, we can gain valuable insights into potential therapeutic strategies for improving jawbone health.

## 2. Mechanisms of Bone Homeostasis in the Jawbone

Bone homeostasis in the jawbone is a complex physiological process regulated by the interaction of various cell types, including bone cells and immune cells [3,15,16]. Unlike other skeletal structures, the jawbone is continuously exposed to mechanical stress and microbial challenges during mastication, requiring a finely tuned balance between bone formation and resorption. In addition to the activity of bone cells, the unique immune microenvironment of the jawbone, characterized by a higher concentration of immune cells, plays a critical role in maintaining homeostasis [2]. This coordinated response to mechanical forces and microbial threats ensures the dynamic regulation of bone remodeling and immune regulation within the jawbone.

### 2.1. Bone Cells in Jawbone Homeostasis

Bone remodeling in the jawbone is a finely tuned process involving the coordinated actions of various bone cells. These cells work together to regulate bone formation and resorption, maintaining skeletal balance.

Mesenchymal cells, such as those in the periodontal ligament, are key regulators of bone remodeling. They are major sources of RANKL, especially during inflammation, driving osteoclast formation and promoting bone resorption [15,17,18]. The RANKL/RANK/OPG axis plays a critical role here: RANKL secreted by mesenchymal cells binds to RANK on osteoclast precursors, promoting osteoclast differentiation and activity. OPG, which acts as a decoy receptor, helps inhibit this process, thus preserving bone mass during non-inflammatory conditions [19]. The balance between RANKL and OPG production by mesenchymal cells is crucial in regulating bone resorption and preventing pathological bone loss [20].

Osteocytes play a central role in bone metabolism by sensing mechanical stress and coordinating the activities of osteoblasts and osteoclasts to maintain homeostasis [4]. They are a significant source of RANKL, especially in response to mechanical unloading or microdamage [15], which drives osteoclast activity. Conversely, osteocytes also produce OPG, which acts as a decoy receptor for RANKL, inhibiting osteoclastogenesis and protecting against excessive bone resorption. The RANKL/OPG balance maintained by osteocytes is essential for regulating bone turnover and adapting to mechanical forces.

Osteoblasts are specialized cells essential for bone formation. They produce the bone matrix, which is primarily composed of collagen, and regulate the mineralization process [5]. Osteoblasts also express RANKL, a key factor in activating osteoclasts, the cells responsible for bone resorption. In inflammatory conditions, RANKL expression by osteoblasts increases, leading to heightened osteoclast activity and accelerated bone resorption [21]. To counterbalance this, osteoblasts produce OPG, which helps reduce bone resorption by inhibiting RANKL’s effects on osteoclasts [22].

Osteoclasts are the key cells responsible for bone resorption, breaking down bone tissue as part of the ongoing bone remodeling process [16]. Their activity is carefully controlled by the RANKL/RANK/OPG signaling pathway [23]. Inflammatory cytokines like tumor necrosis factor (TNF)-α, interleukin (IL)-1β, and IL-6 upregulate RANKL expression, which in turn promotes the formation and activation of osteoclasts, leading to increased bone resorption [14,24,25]. Periodontal ligament stem cells (PDLSCs) play a pivotal role in regulating osteoclast activity through multiple mechanisms, including the secretion of extracellular vesicles (EVs) [26] and apoptotic bodies (ABs) [27], as well as direct cellular interactions. EVs can carry mRNA, proteins, and enzymes that influence osteoclast differentiation and function [28]. ABs contain microRNAs like miR-223-3p, which target integrin beta 1 (Itgb1) to inhibit osteoclast activity and reduce bone resorption [27]. In inflammatory conditions such as periodontitis, PDLSC-derived EVs are enriched with RANKL and TNF-α, promoting osteoclast differentiation and activation [29]. Additionally, PDLSCs regulate osteoclast function through membrane-derived lipid mediators, which are essential for osteoclast morphology and activity [30].

### 2.2. Immune Cells in Jawbone Homeostasis

The jawbone, particularly the alveolar bone, possesses a unique immune microenvironment compared to other bones. This microenvironment is characterized by a higher concentration of mature immune cells that play a crucial role in responding to the constant mechanical and microbial challenges that arise during mastication [2]. These immune responses are essential for the process of bone remodeling, which involves balancing bone resorption and bone formation to adapt to the forces generated by chewing [3].

T cells are pivotal in regulating both bone resorption and regeneration, with different subsets playing distinct roles. RANK, the receptor for RANKL, is expressed not only on osteoclast precursors responsible for bone resorption but also on several immune cells, including T cells [31]. The RANKL/RANK signaling pathway affects the immune system by modulating the development and function of these immune cells. When RANKL binds to RANK on dendritic cells, it enhances their survival and ability to present antigens, which, in turn, activates T cells [32]. In bone metabolism, T helper 1 (Th1) cells produce cytokines such as IL-12 and Interferon (IFN)-γ, which inhibit osteoclast formation by suppressing RANKL and TNF-α pathways, both of which are essential for osteoclast development and activity [33]. On the other hand, T helper 17 (Th17) cells, a subset of CD4+ T cells, promote bone resorption by producing IL-17A, IL-17F, and other cytokines like IL-21 and IL-22 [34]. IL-17 increases RANKL expression on osteoblasts and other bone cells, thereby accelerating osteoclast formation, especially in inflammatory conditions [35,36]. Knocking out IL-17A and IL-17F has been shown to reduce bone loss, underscoring their role in driving inflammation-induced osteolysis [37].

In contrast, regulatory T cells (Tregs) help protect bone by inhibiting excessive immune activity. Through their production of IL-10 and transforming growth factor (TGF)-β, Tregs suppress Th17 cells and reduce RANKL expression, limiting osteoclast formation and bone resorption [11,38,39]. The balance between Th17 and Tregs is influenced by IL-6, which determines whether naive CD4+ T cells develop into pro-resorptive Th17 cells or bone-protective Tregs [40].

γδ T cells, a unique subset of T cells, are distinguished by their specific T-cell receptor (TCR), composed of γ and δ chains. Their role in bone biology is complex, as they produce IL-17A, a cytokine whose effects vary depending on the immune response phase and the surrounding microenvironment [41]. In conditions of chronic inflammation, IL-17A is linked to increased bone resorption. This occurs through the upregulation of RANKL expression on osteoblasts and stromal cells, which enhances osteoclast activation and differentiation, leading to bone breakdown. However, during the early stages of bone healing, γδ T cells’ production of IL-17A supports osteoblast proliferation and activity, promoting bone formation [35].

While certain B cell subsets contribute to bone loss in inflammatory conditions, other B cells can produce anti-inflammatory cytokines like IL-10, which help reduce inflammation and protect bone tissue [11,42,43]. For example, activated B cells in the jawbone significantly contribute to alveolar bone resorption, particularly in response to oral pathogen infections, by producing RANKL [44]. This was demonstrated in a mouse model of periodontitis, where B cell-specific conditional knockout mice revealed the essential role of RANKL-expressing effector B cells in pathogen-induced bone loss [44]. Additionally, B cells interact with osteoclasts and osteoblasts, influencing osteoclast differentiation and activity through the secretion of cytokines such as TNF and C-C chemokine motif ligand (CCL) 3 [45], further contributing to bone loss in inflammatory conditions. On the other hand, regulatory B cells (Bregs) play a crucial role in bone healing by modulating immune responses, particularly by suppressing proinflammatory cytokines and promoting the activity of Tregs [46], thereby helping to reduce inflammation and protect bone tissue.

Macrophages play a crucial role in bone remodeling, with pro-inflammatory macrophages (M1) promoting bone resorption by producing cytokines such as TNF-α, IL-1β, and IL-6, while anti-inflammatory (M2) macrophages support bone regeneration through the secretion of cytokines like TGF-β and IL-10 [11]. The switch from M1 to M2 macrophages is influenced by various factors, including Tregs, which promote the tissue-repairing M2 phenotype [47]. Jaw periosteal cells (JPCs) have been shown to further influence macrophage polarization [48]. In coculture systems, JPCs can induce a shift from M1 to M2 macrophages, promoting an anti-inflammatory environment through paracrine signaling, thereby supporting bone regeneration and tissue repair.

Neutrophils contribute to both bone destruction and repair [49]. During inflammation, they exacerbate bone damage by promoting osteoclast activity through the formation of neutrophil extracellular traps (NETs) and the production of RANKL-inducing cytokines like oncostatin M (OSM) [50]. NETs, formed when neutrophils release their chromatin and antimicrobial proteins into the extracellular space, serve to trap pathogens. However, these NETs also contribute to bone resorption by releasing pro-inflammatory cytokines, such as RANKL, and proteases like elastase, which activate osteoclasts. The formation of NETs triggers the production of reactive oxygen species (ROS), which activate key inflammatory signaling pathways like NF-κB, MAPK, and Janus kinase (JAK)/signal transducer and activator of transcription 3 (STAT3). These pathways, in turn, amplify inflammation, promoting osteoclast differentiation, maturation, and bone resorption. Through the RANKL/RANK signaling axis, NETs enhance osteoclastogenesis by binding RANKL to the RANK receptor on osteoclast precursor cells, leading to increased bone degradation. However, neutrophils are also essential for initiating bone repair during the early stages of the inflammatory response. Additionally, neutrophils are influenced by RANKL, which stimulates their degranulation and migration [32], both essential for immune defense.

Dendritic cells (DCs), along with Langerhans cells, play a crucial role in regulating local immune responses and maintaining bone homeostasis [51]. Through the RANKL/RANK/OPG axis, RANKL enhances the survival and antigen presentation abilities of DCs [31,52], which can influence bone remodeling by activating the immune system. In the jawbone, DCs are particularly important in recognizing bacterial antigens and modulating immune responses [53], essential for maintaining oral health and preventing conditions such as periodontal disease. Additionally, research shows that JPCs can inhibit the maturation of DCs [54], a mechanism that could be beneficial in reducing excessive immune responses during tissue engineering and regenerative procedures.

Innate lymphoid cells (ILCs) play a significant role in both inflammation and tissue repair [55]. In the jawbone, ILC2s promote anti-inflammatory responses by secreting IL-5 and IL-13, which support tissue repair and activate M2 macrophages. They also help maintain the balance between bone resorption and formation [47,56]. Conversely, ILC3s produce IL-17 and IL-22 [57], which contribute to both protective immunity and inflammatory bone resorption.

Moreover, RANKL plays a role beyond immune cell interactions by contributing to the formation and maintenance of lymph nodes, which are central to immune system function [58]. This process is essential for the organization and operation of the immune response, as lymph nodes act as hubs for immune cell activation and interaction.

### 2.3. Mechanisms of Mechanical Stress in Bone Homeostasis

Mechanical stress is a key regulator of bone remodeling, particularly in response to masticatory forces. One of the primary mechanisms involved is fluid shear stress (FSS), which arises from the compressive forces applied to the lacunar–canalicular system during mastication. This shear stress affects osteocytes, which are embedded within the bone matrix and play a pivotal role in mechanotransduction. The compression of the lacunar–canalicular system leads to the activation of intracellular signaling pathways that modulate bone resorption and formation [59].

Mechanoreceptors such as Piezo1 and Piezo2 play a significant role in the response to mechanical loading. These ion channels are activated by tension in the plasma membrane and are crucial for detecting mechanical deformation in cells. Upon activation, Piezo receptors initiate intracellular signaling cascades that influence both bone and immune cell activity, affecting bone remodeling and immune responses [60]. The presence of Piezo1 and Piezo2 mechanoreceptors in various bone and immune cells highlights their essential role in translating mechanical stimuli into biological responses that regulate bone homeostasis and immune regulation [61].

Furthermore, the cytoskeleton plays a crucial role in translating mechanical forces into cellular responses. When cells are subjected to mechanical loading, deformation of the cell membrane occurs, which in turn impacts the structure and function of the cytoskeleton. Focal adhesions and integrins within the cytoskeleton serve as mechanical signal transducers, converting physical forces into biochemical signals. This process is essential for the activation of various signaling pathways, such as the RANKL/RANK/OPG pathway, which regulates osteoclast differentiation and bone resorption [62].

While masticatory forces contribute to the regulation of bone homeostasis, excessive masticatory forces have been shown to cause significant mechanical damage to the jawbone. Overloading the jaw during chewing generates excessive stress on the bone tissue, leading to structural damage such as fractures, bone resorption, or even bone degradation over time [63]. Osteocytes, which are embedded within the bone matrix, are particularly sensitive to such excessive mechanical forces. When masticatory forces exceed the bone’s tolerance, osteocytes detect this damage and activate the bone remodeling process. Osteoclasts are recruited to resorb damaged bone, while osteoblasts are stimulated to generate new bone, repairing and restoring the structural integrity of the jawbone [64].

## 3. The Impact of Masticatory Forces on Immune Cells in the Jawbone

**Figure 1:**
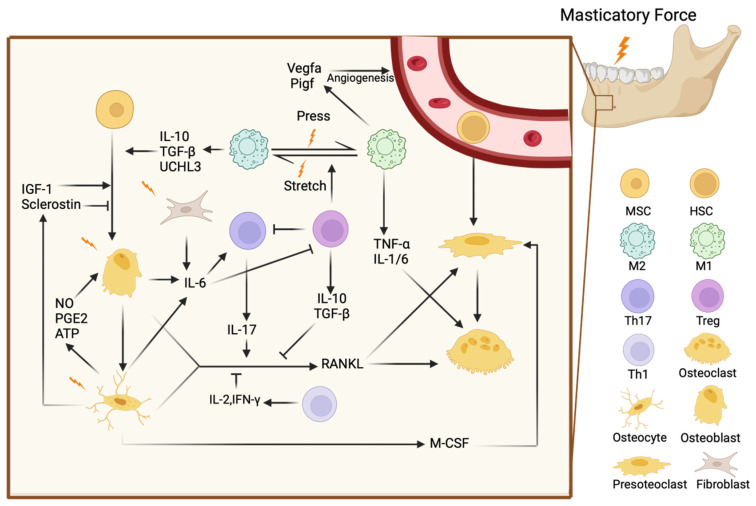
The mandibular immune system and bone cells exhibit a coordinated response under the impact of masticatory forces. Fibroblasts, osteoblasts, and osteocytes secrete IL-6, which leads to subsequent effects. Mechanical forces, such as pressure and stretching, regulate the transition between M1 and M2 macrophages, influencing angiogenesis and osteogenesis. T cells modulate osteoclast activity by regulating the secretion of Rankl. Additionally, the secretions of bone cells affect the differentiation of MSCs and the production of preosteoclasts. M1: M1 macrophages, M2: M2 macrophages, MSC: mesenchymal stem cell, HSC: hematopoietic stem cell.

Masticatory forces not only affect the structural integrity of the jawbone but also play a critical role in influencing immune cell activity, for both innate immune cells and adaptive immune cells [65,66,67] (Figure 1). These forces cause mechanical damage to the gingival barrier, which leads to the release of IL-6 from epithelial cells, fibroblasts, osteoblasts, and skeletal muscle fibers [68]. This IL-6 release promotes the accumulation and function of Th17 cells, which are integral to the immune response in the oral cavity [12]. Additionally, the stiffness of the surrounding environment influences T cell activation and proliferation. Studies using engineered 3D hydrogels have shown that T cells in stiffer environments exhibit greater activation and higher production of cytokines such as IL-2, IFN-γ, and TNF-α compared to softer environments [69].

Mechanical forces also directly activate T cells. Studies using synthetic nanomotors have shown that mechanical forces can open cellular Ca^2+^ channels, triggering T cell activation [70]. This suggests that mechanical stress from mastication could similarly activate T cells in the jawbone, contributing to bone remodeling. Mechanical stress further enhances the expression of T cell-associated Th1 cytokines such as TNF-α and IFN-γ within periodontal tissues, promoting osteoclast activation and facilitating alveolar bone remodeling [65]. Inhibiting TNF-α has been shown to reduce the stimulatory effects of T cells, further underscoring the role of these cytokines in T cell-mediated bone remodeling.

In orthodontic tooth movement (OTM), mechanical forces increase the ratio of CD4+/CD3+ T cells in the alveolar bone, correlating with elevated expression of Th1 cytokines like TNF-α and IFN-γ, which promote bone resorption [64]. Similarly, γδ T cells produce IL-17A, which recruits monocytes and neutrophils, driving osteoclast activation [67]. Since both orthodontic and masticatory forces apply mechanical stress to the jawbone, mastication likely influences T cell activity in a similar way.

Macrophages, which are crucial for bone remodeling, respond to mechanical forces such as those generated during chewing by regulating the activity of osteoclasts and osteoblasts [66]. These mechanical forces influence macrophage polarization, shifting them between M1 and M2 states. Masticatory forces, akin to mechanical stretching, promote the polarization of M2 macrophages, which are essential for bone regeneration [71]. Mechanical stimuli, such as shear stress, compression, and tensile forces, are detected by macrophages through mechanosensors like integrins, ion channels, and other mechanoreceptors. These mechanosensitive molecules translate mechanical signals into biochemical signals, activating intracellular pathways that dictate macrophage function. When macrophages are exposed to mechanical forces, they initiate various signaling cascades. For example, shear stress activates the ERK1/2 signaling pathway in PDLSCs, which enhances the secretion of immunomodulatory molecules such as TGF-β1 and IL-10 [72,73,74,75]. These molecules promote the M2 polarization of macrophages. Additionally, integrins play a central role in mechanosensing by linking the extracellular matrix to the intracellular cytoskeleton. The activation of integrins in response to mechanical forces leads to the activation of downstream signaling pathways, including the NF-κB and MAPK pathways, which are critical in modulating the immune response and macrophage polarization. The mechanical forces also affect macrophage polarization indirectly through the release of extracellular vesicles, such as exosomes, from activated macrophages. These exosomes contain bioactive molecules, including ubiquitin carboxyl-terminal hydrolase isozyme L3 (UCHL3) [75], that further promote osteogenesis and contribute to the regeneration of alveolar bone. Furthermore, the mechanical environment influences macrophage behavior through the modulation of the extracellular matrix (ECM). In response to mechanical stress, the ECM undergoes remodeling, which in turn affects macrophage function. This remodeling process can provide physical cues that guide macrophage differentiation toward the M2 phenotype, further supporting tissue regeneration and bone homeostasis. M2 macrophages enhance osteogenesis in bone marrow mesenchymal stem cells (BMSCs) by producing anti-inflammatory factors like IL-10 and TGF-β1 [72,73,74,75]. Additionally, chewing stimulates the release of exosomes from activated macrophages, which contain factors such as ubiquitin carboxyl-terminal hydrolase isozyme L3 (UCHL3) [75,76]. These exosomes further promote osteogenesis in BMSCs and contribute to alveolar bone formation. This process demonstrates how macrophages, through both direct cytokine production and exosome-mediated signaling, respond to mechanical stress to support bone regeneration and maintain bone health. M2 macrophages also contribute to angiogenesis by increasing the expression of growth factors such as vascular endothelial growth factor (Vegfa) and placental growth factor (Pigf), promoting effective bone remodeling [73].

The Piezo1 ion channel, a key mechanosensor in macrophages, is upregulated by mechanical forces such as mastication, activating the protein kinase B (AKT)/glycogen synthase kinase 3 β (GSK3β) signaling pathway, which promotes macrophage proliferation and supports bone regeneration [72]. During orthodontic tooth movement, compressive forces also activate the NOD-like receptor family pyrin domain containing 3 (NLRP3) inflammasome, driving osteoclast differentiation and alveolar bone resorption [77]. Given that chewing exerts similar mechanical stress on the jawbone, it is likely that mastication influences these pathways in a similar manner.

Finally, interactions between immune cells, particularly monocytes and macrophages, and MSCs are more pronounced in the jawbone compared to in long bones. This unique immune microenvironment underscores the complex communication between immune cells and bone cells in response to mechanical forces [2].

## 4. The Regulatory Effects of Masticatory Forces on Bone Cells in the Jawbone

Masticatory forces play a critical role in regulating bone remodeling by activating mechanotransduction pathways in osteocytes, osteoblasts, and osteoclasts. These mechanical signals influence both bone formation and resorption, ensuring the jawbone adapts to the functional demands of chewing while maintaining structural integrity (Figure 1).

### 4.1. Mechanotransduction and Immune Response in Osteocytes

Mastication plays a critical role in regulating bone formation and resorption through its influence on osteocyte activity, which is key to maintaining jawbone health. Osteocytes, the most abundant bone cells, are crucial for mechanosensation—a process where these cells detect mechanical forces such as those produced by chewing [7,8,9]. Upon detecting mechanical loading, osteocytes activate gene expression and produce proteins that contribute to bone remodeling, either promoting bone formation or triggering bone resorption [78].

The mechanotransduction process in osteocytes is triggered by mechanical stress, leading to plasma membrane disruptions (PMDs), activation of integrin-based structures, and involvement of the lacunar–canalicular system (LCS). Studies by Yu Kanglun et al. demonstrate that mechanical loading disrupts the plasma membranes of osteocytes, which initiates calcium signaling and other pathways that promote bone adaptation [10]. This response is further supported by specialized structures like β3 integrin sites on osteocyte processes, which are particularly sensitive to mechanical strain, allowing osteocytes to effectively sense and respond to these forces [79]. Mastication also exerts mechanical forces such as compression, stretching, and bending on the jawbone, creating FSS in the intraosseous fluid within the LCS [80,81,82]. This process is crucial for osteocytes to sense mechanical load. Osteocytes, connected through dendrites to form a complete network within the bone matrix, detect FSS and other stimuli, including matrix stiffness and hydrostatic pressure [8,83,84]. These mechanical signals are transmitted via protein secretion and cell dendrites [85], contributing to bone remodeling. Further computational research suggests that the poroelastic properties of bone cells significantly affect their response to compressive loads, influencing both strain distribution and fluid flow within the bone matrix [86].

Kawakami et al.’s studies revealed that masticatory forces affect osteocyte micromorphology and bone lacunae characteristics. For example, rats fed a solid diet developed more complex osteocyte connectivity and larger bone lacunae compared to those on a powdered diet [85]. This indicates that chewing increases the structural complexity of osteocytes and enhances bone lacunae expansion. Additionally, Dentin matrix protein (DMP)-1, an important marker for osteocyte activity and bone calcification, showed higher localization in the solid feed group, suggesting that mastication significantly influences bone calcification [85].

The Wingless/Int-1 (Wnt)/β-catenin and Yes-associated protein (YAP)/Transcriptional co-activator with PDZ-binding motif (TAZ) pathways are both pivotal in the regulation of bone remodeling and mechanotransduction in osteocytes under mechanical loading. The Wnt/β-catenin pathway is central to maintaining bone mass, as its activation promotes osteoblastogenesis [87]. However, this pathway can be impaired by factors such as estrogen deficiency, as observed in ovariectomized mouse models [88]. Similarly, the YAP/TAZ pathway is activated by mechanical loading and regulates the expression of mechanosensitive genes and chemokines, which are essential for bone remodeling [89].

Further research has shown that increased masticatory activity, such as chewing a hard diet, suppresses sclerostin, a protein that inhibits bone formation, and induces the expression of Insulin-like growth factor (IGF)-1 in osteocytes [90]. IGF-1 receptor expression is also upregulated in chondroblasts due to enhanced muscle activity during mastication [91], which leads to thickening of the articular cartilage and promotes jawbone development [90]. Mechanical loading also stimulates the production of IL-6 in osteocytes, which activates JAK/STAT3 and extracellular signal-regulated kinase (ERK) signaling pathways. These pathways promote bone formation while inhibiting bone resorption [92,93].

Mastication further influences the immune environment of the jawbone by upregulating chemokines like C-X-C motif chemokine ligand (CXCL) 1 and CXCL2 in response to mechanical forces [94]. These chemokines promote the proliferation of bone cells, while other signaling molecules, such as macrophage colony-stimulating factor (M-CSF), CXCL1, CXCL2, CXCL3, CXCL9, and CXCL10, are expressed through the YAP/TAZ pathway, playing a significant role in osteocyte mechanotransduction and overall bone health [89]. Additionally, mechanical loading stimulates osteocytes to release nitric oxide (NO), prostaglandins (PGE2), and adenosine triphosphate (ATP), increasing the expression of osteogenic markers such as osterix (OSX) and alkaline phosphatase (ALP), which enhance osteoblast differentiation and bone mineralization [95,96].

Studies by Lohberger et al. show that cyclic mechanical stimulation, similar to that experienced during chewing, increases the expression of osteogenesis-specific markers, including type-I collagen and bone morphogenetic protein-2 in human intraoral mesenchymal stromal and progenitor cells [97]. This highlights the direct impact of mechanical forces, like mastication, on bone cell function and development.

### 4.2. Mechanotransduction and Immune Response in Osteoblastes

Mechanical stress significantly influences jawbone metabolism by affecting the activities of osteoblasts, the cells responsible for bone formation, and osteoclasts, which are involved in bone resorption. Specifically, mechanical stress enhances osteoblast activity through pathways involving key proteins such as glucose transporter (Glut) 1, sirtuin (SIRT) 1, and the transcription factor Runt-related transcription factor (Runx) 2, leading to increased bone formation [98,99].

Increased masticatory force activates osteocytes, the bone cells that act as mechanosensors. These activated osteocytes modify the expression of signaling molecules to promote the formation of osteoblasts. Specifically, they upregulate IGF-1 and suppress sclerostin, thereby enhancing the differentiation of osteoblasts from precursor cells [90].

Intermittent compressive force also stimulates osteoblast differentiation through the Wnt/β-catenin signaling pathway. This process is mediated by the release of ATP from the cells, which increases the expression of genes involved in bone formation and promotes mineralization [96]. Additionally, mechanical stress activates enzymes called matrix metalloproteinases (MMPs), particularly MMP-2, MMP-13, and MT1-MMP [100]. These enzymes are essential for osteoblast differentiation, facilitating the expression of important bone formation markers and contributing to the mineralization of the bone matrix.

Masticatory force also influences osteoblastic activity in the jaw by altering the expression of key signaling molecules such as RANKL and OPG. Mechanical stress shifts the balance toward increased RANKL expression, promoting bone remodeling through the formation of osteoclasts in a process known as osteoclastogenesis. This leads to bone resorption by osteoclasts [101], followed by new bone formation by osteoblasts [102]. Furthermore, mechanical stress enhances RANKL expression via the p38 MAPK pathway, underscoring the essential role of chewing forces in regulating osteoblast activity and maintaining bone health in the jaw [103,104].

Osteoblasts respond differently depending on the magnitude of compressive stress applied. At optimal stress levels, osteoblast differentiation is enhanced, evidenced by increased levels of bone formation markers such as Runx2 and ALP. However, excessive stress can inhibit the ability of osteoblasts to regulate the formation of osteoclasts [105].

### 4.3. Mechanotransduction and Immune Response in Osteoclastes

Mechanical forces, such as masticatory force, regulate osteoclast activity through a signaling pathway mediated by cementocytes. Under force loading, cementocytes facilitate osteoclastogenesis via the sphingosine-1-phosphate (S1P)/sphingosine-1-phosphate receptor (S1PR) 1/ras-related C3 botulinum toxin substrate 1 (Rac1) axis [106,107,108,109], where increased compression leads to higher synthesis and release of S1P, amplifying RANKL production and promoting osteoclast differentiation.

Moreover, masticatory force also exerts significant influence on osteoclast differentiation and macrophage activity through the actions of PDLSCs [110,111,112,113]. Under mechanical force, PDLSCs secrete exosomes that modulate immune responses, notably by suppressing IL-1β production through inhibition of the NF-κB signaling pathway in macrophages [112]. This mechanical stimulation also alters the exosomal proteome, increasing levels of annexin A3 (ANXA3), which enhances exosome uptake and activates the ERK pathway, promoting osteoclast differentiation [114]. Furthermore, force-induced production of hydrogen sulfide (H2S) by PDLSCs is associated with the secretion of monocyte chemoattractant protein (MCP)-1 and regulation of the RANKL/OPG system, both of which are critical for macrophage migration and osteoclast differentiation, key processes in bone remodeling [115,116,117].

In addition, masticatory force enhances osteoclastogenesis by bone marrow macrophages (BMMs) through the activation of colony-stimulating factor 1 receptor (CSF1R) signaling [118]. This process promotes osteoclast differentiation induced by TNF-α in the presence of M-CSF, independent of RANKL [119,120]. Notably, this highlights the crucial role of compressive force in driving osteoclastogenesis, even in the absence of mechanosensitive cells such as osteoblasts and fibroblasts.

While mechanical forces can promote osteoclastogenesis through enhanced RANKL production, other studies have demonstrated that increased masticatory force can actually diminish osteoclast activity by modulating the RANKL/OPG ratio. Specifically, increased masticatory force influenced the expression of essential proteins within the alveolar bone. Notably, the experimental group exhibited a substantial rise in OPG and mechano-growth factor (MGF) levels, alongside a marked reduction in RANKL. This led to a decreased RANKL/OPG ratio, diminishing osteoclast activity, which can help in maintaining or increasing bone density and is advantageous for bone health [121].

## 5. Broader Impacts of Masticatory Forces on Jawbone Immunity and Remodeling

**Figure 2:**
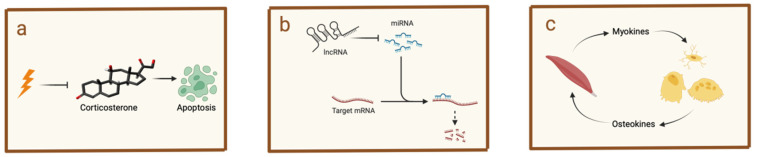
Masticatory forces influence jawbone immunity and bone balance through other pathways. Masticatory forces can influence jawbone immunity and bone balance by (**a**) regulating hormone levels, (**b**) affecting non-coding RNA, and (**c**) modulating muscle-bone interaction.

Recent studies have revealed that masticatory forces influence the jawbone’s immune environment and bone remodeling not only through direct mechanical stimuli but also through hormonal interactions, non-coding RNA regulation, and muscle-bone communication (Figure 2). These mechanisms highlight the complexity of how chewing affects overall jawbone health, extending beyond basic bone cell activity to include broader systemic effects.

### 5.1. Hormonal Modulation of Jawbone Immunity by Masticatory Forces

Recent studies have shown that chewing affects immune function by altering hormone levels [122]. During fasting, leptin, a hormone that regulates immune cell activity [123], typically decreases. Meanwhile, corticosterone, a stress hormone known to suppress immune cell growth and induce cell death [124], increases. Research by Yang et al. found that chewing stimulation in fasting mice reduced the rise in corticosterone levels without affecting the decline in leptin. This suggests that chewing can mitigate stress-related immune suppression. Furthermore, chewing was observed to enhance antibody production after immunization, indicating that it may strengthen immune responses under fasting conditions by counteracting the effects of stress hormones [125].

### 5.2. Influence of Masticatory Forces on Jawbone Immunity Through Non-Coding RNA Regulation

Mechanical stress from chewing has been shown to influence bone remodeling by regulating small RNA molecules that control gene expression. MicroRNAs (miRNAs) are small, non-coding RNA molecules that regulate genes after transcription by binding to target messenger RNAs, leading to their degradation or inhibition of translation. They play critical roles in various biological processes, including bone remodeling [1].

Under compressive forces, specific miRNAs are either increased or decreased to mediate bone cell responses. For example, miR-494-3p levels increase under compressive stress and inhibit the proliferation of osteoblasts by targeting key genes essential for bone differentiation, such as *FGFR2* and *ROCK1* [126]. Similarly, miR-29 regulates components of the extracellular matrix, affecting bone matrix formation [127]. These miRNAs help balance the activity between osteoblasts and osteoclasts, driving bone resorption and remodeling in response to mechanical forces.

Mechanical stress also affects bone remodeling through long non-coding RNAs (lncRNAs), which are longer RNA molecules that do not code for proteins but regulate gene expression [128]. In response to mechanical stimuli like compressive forces, the lncRNA H19 is upregulated and plays a crucial role in the differentiation of mesenchymal stem cells into bone cells [129]. H19 primarily influences the Notch signaling pathway, essential for bone development and remodeling. Any imbalance in H19 expression can disrupt normal bone formation, highlighting the important regulatory function of lncRNAs in maintaining proper bone remodeling dynamics.

These findings underscore the complexity of how mechanical stress from chewing influences the immunological environment of the jawbone. By regulating non-coding RNAs like miRNAs and lncRNAs, mechanical forces modulate gene expression, affecting bone remodeling and immune responses in the jaw.

### 5.3. Muscle–Bone Interaction Mediated by Masticatory Forces

Over the past decade, the concept of muscle–bone crosstalk has gained significant attention. This interaction encompasses not only the mechanical forces generated during muscle contraction but also biochemical signals mediated by soluble molecules [130]. The masseter muscle, a primary muscle involved in chewing, has been identified as both a source of mechanical force affecting the jawbone’s immune environment through mechanotransduction and as a secretory organ influencing mandibular bone osteoimmunology [131,132].

In the mandible, this interaction is particularly crucial due to the close anatomical relationship between the masseter muscle and the jawbone. Muscle-derived signaling molecules, known as myokines, play a key role in communication between skeletal muscle and bone. These myokines include myostatin, brain-derived neurotrophic factor (BDNF), irisin, IGF, fibroblast growth factor (FGF)-2, β-aminoisobutyric acid (BAIBA), and several interleukins such as IL-6, IL-7, IL-8, and IL-15 [133,134,135,136]. For example, myostatin directly influences bone cell behavior by promoting the formation of osteoclasts, the cells responsible for bone resorption, through RANKL signaling. It also inhibits the differentiation of osteoblasts, the cells responsible for bone formation, by altering the content of exosomes released from osteocytes, which are mature bone cells [137,138]. Additionally, IGF-1, produced in skeletal muscle, is significantly increased following exercise [139]. This increase enhances muscle–bone interaction and affects the expression of osteoclasts and osteoblasts [140].

Conversely, bone-derived signaling molecules, known as osteokines, contribute to this bidirectional communication. Osteokines such as RANKL, osteocalcin, sclerostin, PGE2, TGF-β, Wnt3a, and FGF-23 play roles in regulating both local bone processes and systemic physiological functions. These molecules further emphasize the complexity of muscle–bone interactions and their impact on overall skeletal health [141,142,143,144].

## 6. Conclusions

In summary, the intricate interplay between mastication and the immune environment of the jawbone represents a critical focus within the field of osteoimmunology. This review underscores the complex mechanisms through which masticatory forces drive bone remodeling and modulate immune responses, with particular emphasis on the roles of key bone cells and immune cells. Central to this process is the RANKL/RANK/OPG signaling pathway, which regulates the balance between bone formation and resorption in response to mechanical stress. Moreover, masticatory forces exert systemic effects, influencing immune regulation through hormonal pathways and the modulation of non-coding RNAs. A deeper understanding of these interactions enhances our comprehension of jawbone health and offers potential avenues for the development of targeted therapies for conditions such as periodontal disease.

However, despite the insights provided, several challenges remain, such as the detrimental effects of excessive masticatory forces, which can lead to bone damage and inflammation. Addressing these issues requires further exploration into the specific molecular pathways involved and the identification of effective preventive or therapeutic interventions. For instance, developing mechanical force-modulating devices or exploring pharmacological approaches that target key signaling molecules within the RANKL/RANK/OPG pathway could offer solutions to mitigate excessive bone resorption or enhance bone formation. Additionally, the role of non-coding RNAs in immune modulation presents a promising area for the development of novel treatments. By advancing research in these areas, we may uncover strategies for preserving bone integrity and immune homeostasis in the jaw, contributing to better management of periodontal disease and other related conditions.

## Data Availability

No new data were created or analyzed in this study.

## Abbreviations

RANKL	receptor activator of nuclear factor κ-B ligand
RANK	receptor activator of nuclear factor κ-B
OPG	osteoprotegerin
TNF	tumor necrosis factor
IL	interleukin
PDLSCs	periodontal ligament stem cells
EVs	extracellular vesicles
ABs	apoptotic bodies
Itgb1	integrin beta 1
Th1	T helper 1 cells
IFN	interferon
Th17	T helper 17 cells
Tregs	regulatory T cells
TGF	transforming growth factor
TCR	T-cell receptor
CCL	C-C chemokine motif ligand
M1	pro-inflammatory macrophages
M2	anti-inflammatory macrophages
JPCs	jaw periosteal cells
NETs	neutrophil extracellular traps
ROS	reactive oxygen species
OSM	oncostatin M
DCs	dendritic cells
ILCs	innate lymphoid cells
OTM	orthodontic tooth movement
BMSCs	bone marrow mesenchymal stem cells
UCHL3	ubiquitin carboxyl-terminal hydrolase isozyme L3
Vegfa	vascular endothelial growth factor
Pigf	placental growth factor
AKT	protein kinase B
GSK3β	glycogen synthase kinase 3 β
NLRP3	NOD-like receptor family pyrin domain containing 3
PMD	plasma membrane disruptions
LCS	lacunar-canalicular system
FSS	fluid shear stress
DMP	dentin matrix protein
Wnt	Wingless/Int-1
YAP	yes-associated protein
TAZ	transcriptional co-activator with PDZ-binding motif
IGF	insulin-like growth factor
JAK	janus kinase
STAT3	signal transducer and activator of transcription 3
ERK	extracellular signal-regulated kinase
CXCL	C-X-C motif chemokine ligand
M-CSF	macrophage colony-stimulating factor
NO	nitric oxide
PGE2	prostaglandins
ATP	adenosine triphosphate
OSX	osterix
ALP	alkaline phosphatase
Glut	glucose transporter
SIRT	sirtuin
Runx	runt-related transcription factor
S1P	sphingosine-1-phosphate
MMPs	matrix metalloproteinases
Rac1	ras-related C3 botulinum toxin substrate 1
ANXA3	annexin A3
H2S	hydrogen sulfide
MCP	monocyte chemoattractant protein
BMMs	bone marrow macrophages
CSF1R	colony-stimulating factor 1 receptor
MGF	mechano-growth factor
miRNAs	microRNAs
lncRNAs	long non-coding RNAs
BDNF	brain-derived neurotrophic factor
FGF	Fibroblast growth factor
BAIBA	β-aminoisobutyric acid
